# Tactical deception to hide sexual behaviour: macaques use distance, not visibility

**DOI:** 10.1007/s00265-015-1946-5

**Published:** 2015-06-05

**Authors:** A. M. Overduin-de Vries, B. M. Spruijt, H. de Vries, E. H. M. Sterck

**Affiliations:** Utrecht University, Utrecht, The Netherlands; Biomedical Primate Research Centre, Rijswijk, The Netherlands

**Keywords:** Cognition, Tactical deception, Sneaky mating, Hiding, Macaque, Old world monkey

## Abstract

Although tactical deception (TD) may be employed to hide sexual behaviour, there is as yet no firm evidence for it. Hiding may be guided by cognitive mechanisms consistent with either no, low or high level TD, such as exploiting male peripheral positions (no TD), creating distance (TD level 1) or hiding behind screens (TD level 1.5 which involves visual perspective taking (VPT)). Macaques are capable of VPT in a food context, suggesting that they may employ TD level 1.5. We investigated, in an observational study with temporarily provided hiding screens, which strategy was used to hide sexual behaviour in captive groups of two macaque species (*Macaca mulatta* and *Macaca fascicularis*). Sexual behaviour only sporadically took place near screens, and the few copulations near screens were not systematically hidden from the alpha male, precluding TD level 1.5. Instead, both females and non-alpha males were at a larger distance from the alpha male during sexual interactions than otherwise, consistent with TD level 1. Creating peripheral locations (TD level 1) may be effective in improving sexual opportunities in many species.

**Electronic supplementary material:**

The online version of this article (doi:10.1007/s00265-015-1946-5) contains supplementary material, which is available to authorized users.

## Introduction

Social complexity is considered an important selective force driving animal intelligence, as proposed in the social intelligence hypothesis (Jolly 1966; Humphrey 1976; Byrne and Whiten 1988; Dunbar 1998). Some animal species, such as primates, cetaceans, carnivores, elephants and corvids, live in relatively complex social systems characterized by a high degree of fission-fusion dynamics and long-term bonds comprising both cooperative and competitive relationships (Amici et al. 2008; Bugnyar 2013). This constant confrontation with social problems may have driven the evolution of complex cognitive capacities (Emery and Clayton 2004). One complex social strategy is tactical deception (TD), for which three levels are distinguished. In the most basic level, TD level 1, ‘an actor employs a signal or action in an atypical context, thereby disadvantaging a misinformed competitor to the benefit of the actor’ (Byrne and Whiten 1990, p. 2; le Roux et al. 2013). TD level 1 may be achieved by learning a specific behaviour that often results in deception with a benefit for the deceiver (i.e. operant conditioning). However, deception is not considered tactical if the signal or action is a coincident rather than a signal or action with the goal of benefitting from the deception (Brockman 1999). TD level 1.5 adds to the definition of TD level 1 an understanding of what other individuals can see, i.e. visual perspective taking (VPT), and level 2 adds to level 1 an understanding of deception, where the actor attempts to manipulate the knowledge of the other individual (following Byrne and Whiten 1990). Evidence of TD has been found for fish (cleaner wrasses, *Labroides dimidiatus*: Bshary 2002; Soares et al. 2014), corvids (ravens, *Corvus corax*: Bugnyar and Heinrich 2006), apes (chimpanzees, *Pan troglodytes*: Melis et al. 2006) and monkeys (brown capuchin monkeys, *Cebus apella*: Fujita et al. 2002; Amici et al. 2009; rhesus monkeys, *Macaca mulatta*: Santos et al. 2006; brown lemurs, *Eulemur fulvus*: Genty et al. 2008; long-tailed macaques, *Macaca fascicularis* and spider monkeys, *Ateles geoffroyi*: Amici et al. 2009).

TD in corvids (Bugnyar and Heinrich 2006) and apes is found up to level 2 (Hare et al. 2006). In contrast, for most of the fish (Bshary 2002) and monkeys (Fujita et al. 2002; Santos et al. 2006; Genty et al. 2008; Amici et al. 2009), tactical behaviour is based on operant conditioning, or monkey tactical behaviour was only observed in the case of one individual and the evidence is not convincing (Fujita et al. 2002). Therefore, evidence for TD in fish and monkeys is most congruent with TD level 1 and not based on an understanding of the visual perspective of another individual (TD level 1.5) nor of the deceptive situation (TD level 2).

One of the contexts in which TD may be used by primates is sneaky mating (Byrne and Whiten 1990). In many primate groups, high-ranking males typically have priority of access to fertile females (Altmann 1962; Cowlishaw and Dunbar 1991), yet in other groups no such priority of access is found (McMillan 1989; de Ruiter et al. 1992; Alfaro 2005; Dubuc et al. 2011; Massen et al. 2012). For rhesus macaques, both positive (Altmann 1962; Cowlishaw and Dunbar 1991) and negative results (Dubuc et al. 2011; McMillan 1989; Massen et al. 2012) exist, depending on the group studied. For long-tailed macaques, there is only one study with negative results (de Ruiter et al. 1992). Non-alpha males and females can copulate sneakily to counter the monopolization of females by dominant males (hamadryas baboon, *Papio hamadryas*: Kummer 1968; long-tailed macaques: Gygax 1995; Kummer et al. 1996; Overduin - de Vries et al. 2013; geladas, *Theropithecus gelada*: le Roux et al. 2013; rhesus macaques: Overduin - de Vries et al. 2012). Female and non-alpha male macaques copulate more often if specific individuals, in particular higher-ranking males, are out of sight (rhesus macaques: Ruiz de Elvira and Herndon 1986; Overduin - de Vries et al. 2012; long-tailed macaques: Gygax 1995; Overduin - de Vries et al. 2013). An important question that remains unanswered by these studies is at what cognitive level hiding is achieved and what level of TD is employed.

Hiding sexual behaviour may result from six different behavioural strategies, which range from basic (no TD) to complex (with TD) (Gygax 1995), as described in the following.

- No tactical deception
  1. *Exploiting peripheral locations*. Sexual behaviour takes place at locations distant from high-ranking males. Peripheral positioning of non-alpha males results from non-sexual behaviour, such as fleeing or avoidance behaviour (Hemelrijk 1998, 2000; Evers et al. 2011, 2012). It is the females who are willing to copulate that approach these peripheral non-alpha males (rhesus macaques: Kaufmann 1965; Drickamer 1974; Berard et al. 1994; Dubuc et al. 2011; Japanese macaques, *Macaca fuscata*; Inoue and Takenaka 2008). This strategy does not involve TD, because the goal of the peripheral positioning is not to gain the benefits from hiding sexual behaviour.
- Tactical deception level 1.0
  2. *Creating peripheral locations*. In this case, both individuals willing to copulate actively increase their distance from specific bystanders, e.g. alpha males. Copulations farther away from specific bystanders are less likely to be disturbed (le Roux et al. 2013); moreover, individuals inhibit their sexual behaviour in the proximity of certain bystanders, resulting in an audience effect (Overduin - de Vries et al. 2012, 2013).
  3. *Sexual behaviour near opaque objects*. Sexual behaviour is performed close to opaque objects, e.g. screens, irrespective of whether the bystander is on the same side of the object or not. This strategy may result from having experienced less aggression from occasional copulations near opaque objects.
  4. *Sexual behaviour behind opaque objects*. Both individuals position themselves on one side of an opaque object, while the audience is on the opposite side. This strategy may result from the rule ‘avoid seeing the audience while involved in sexual behaviour’.
    Strategies 2–4 may be learned by operant conditioning. Although these strategies do not necessarily involve true hiding, they make sexual behaviour less conspicuous and therefore may lead to deception of the alpha male (Byrne and Whiten 1990; le Roux et al. 2013). Deception achieved using one of these strategies can be considered TD level 1.0 if an individual repeatedly uses the strategy and if the tactical behaviour is specifically linked to obtaining benefits from concealed copulation.
- Tactical deception level 1.5
  5. *True intentional hiding*. Both individuals hide themselves, including distal body parts, while occasionally monitoring the audience through peek-holes. This strategy involves TD level 1.5, since an individual has to discriminate its own visual perspective from the perspective of the audience.
- Tactical deception level 2.0
  6. *Intentional deception*. Before completely hiding themselves, as in the fifth strategy, the individuals make use of indirect approaches to opaque objects (i.e. using detours), concealing their intention to hide (cf. chimpanzees: Hare et al. 2006); they only do this, however, when the audience is watching their movements.

All six strategies may effectively hide sexual behaviour, even if in the first three strategies the individuals may not be (completely) hidden from bystanders. The fourth strategy will be more effective, but requires learning a slightly more complex rule than the first three strategies. However, the most effective strategies are the fifth and sixth ones, but these require respectively VPT (TD level 1.5) and theory of mind (TD level 2), which are even more cognitively demanding.

There is some evidence that long-tailed macaques possess VPT, a requirement for TD level 1.5 (Goossens et al. 2012; Overduin - de Vries et al. 2014). However, there is little evidence that these macaques use VPT in hiding behaviour. Inconclusive evidence comes from an experiment with opaque screens: the long-tailed macaques at first tended to hide their sexual behaviour behind the screens, but then stopped doing so, probably because they realised that the wire mesh present prevented the alpha male from disrupting their copulations (unpublished experiment mentioned in Kummer et al. 1996). In another study, where the alpha male was present in the group, low- and middle-ranking long-tailed macaque males preferred to copulate near opaque structures (Gygax 1995). However, low-ranking males also preferred being in the vicinity of opaque structures during non-sexual events. Moreover, it is not clear what property of the screens elicited this preference: restricted visibility, restricted accessibility, elevated locations or peripheral locations.

To evaluate which behavioural strategies macaques use to hide sexual behaviour, we studied two different species of macaques: rhesus and long-tailed macaques. The more despotic rhesus alpha males may be stricter in behaving aggressively towards copulating non-alpha males than the long-tailed alpha males (Thierry 2007). This may predict that rhesus macaques are more likely to hide copulations than long-tailed macaques. Yet, rhesus macaques are multiple mounters, whereas long-tailed macaques often display single-mount copulations (Shively et al. 1982). Since single-mount copulations are shorter than multiple-mount copulations, this may predict that long-tailed macaques will more readily use opaque objects to hide copulations than rhesus macaques. In any event, in both species non-alpha males and females have incentives to hide sexual behaviour.

The spatial location and inter-individual distances between the monkeys were recorded during sexual and non-sexual events. We predicted that if *creating peripheral locations* (TD level 1) is used as a strategy, the inter-individual distances during sexual events would be larger than during non-sexual events, whereas if *exploiting peripheral locations* (no TD) is used, there would be no such difference. The groups were provided with several screens, which varied in terms of the size of the surface that was opaque, accessibility and the presence or not of peek-holes; these variations allowed for a discrimination between strategies 3, 4, 5 and 6. We predicted that if *sexual behaviour near opaque objects* (TD level 1) is used, monkeys would copulate more often within 2 m of a screen than would be expected, based on the available space near or away from screens. If *sexual behaviour behind opaque objects* (TD level 1) is used, we would further expect the monkeys to copulate more often behind than in front of an opaque object, looked at from the perspective of the alpha male. For strategies 5 and 6, no specific predictions were tested, but we would at least expect that if one of these strategies is used, monkeys would copulate more often behind than in front of an opaque object, looked at from the perspective of the alpha male. If we were to find this, this would warrant a follow-up study to discriminate between strategies 4, 5 and 6.

## Methods

### Subjects

Two groups of rhesus macaques (group 1, ‘Lewinsky’, and group 2,’Bram’) and two groups of long-tailed macaques (group 3, ‘Lixa’, and group 4, ‘Tofa’) were studied. Groups 1 to 4 included 10, 10, 15 and 16 adult females, respectively, and 7, 4, 7 and 5 adult males. Only sexually mature monkeys, i.e. females older than 3.5 years and males older than 3 years, were included in the analysis. All males were intact, and the age of the non-alpha male subjects ranged between 3 and 26 years (*N* = 19, mean = 7.7, SD = 5.7) comprising the prime time of macaque males between their 6th and 11th years (Bercovitch et al. 2003). All individuals had several potential non-kin (i.e. from another matriline) sexual partners in the group. Each subject contributed to the dataset as a potential part of a sexual dyad and as a possible bystander individual. Within each group, the hierarchy was determined based on submissive behaviours using Matman 1.1 (de Vries et al. 1993). In all four groups, the dominance hierarchy was significantly linear [groups 1 to 4, respectively: h’ = 0.53, 0.50, 0.55, 0.82, *p* = 0.0006, 0.0001, 0.0004, 0.0001 (de Vries 1995)]. Rhesus macaques are seasonal breeders, and their mating season in our colony lasts from October to March. Long-tailed macaques may be seasonal breeders (van Schaik and van Noordwijk 1985), yet in our colony are non-seasonal and mate all year round.

### Housing

The rhesus and long-tailed macaques were housed in similar cages. The cages consisted of interconnected indoor and outdoor enclosures. Enclosures were provided with permanent enrichment consisting of fire hoses, tires, ladders, tree trunks and a swimming pool (Vernes and Louwerse 2010). Monkeys were only fed in the indoor enclosure, outside observation hours. However, water was available ad libitum in the outdoor enclosure.

**Fig. 1:**
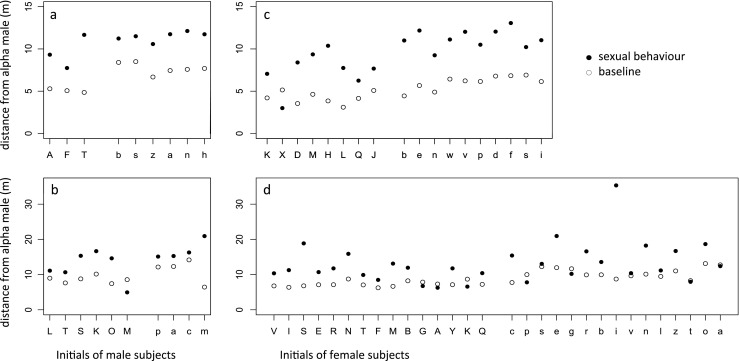
The mean distance of each adult individual from the alpha male during sexual behaviour (*black dots*) and baseline (*open dots*), for rhesus macaque **a** males and **b** females, and long-tailed macaque **c** males and **d** females. Each panel includes two groups; groups are separated by an *open slot*; *single letter codes* for individuals are provided, with *capital letters* for groups 1 (**a**, **b**) and 3 (**c**, **d**), and *lowercase letters* for groups 2 (**a**, **b**) and 4 (**c**, **d**). Individuals are ordered from high (*left*) to low (*right*) dominance rank

During the observations, only the outdoor enclosures were available to the monkeys. The outdoor enclosures measured for groups 1 to 4: 260, 270, 218 and 502 m^2^, respectively, and were of approximate triangular shape (Fig. 1 in Overduin - de Vries et al. 2012, 2013). Compared to groups 1, 2 and 3, group 4 had access to a double-sized outside enclosure (i.e. group 4 used two connected outdoor enclosures).

### Observations

The groups were observed by five different observers (because of its larger outdoor area, group 4 was observed by two observers simultaneously). The groups were observed over different periods: group 1, 25-11-2008 to 17-02-2009; group 2, 25-11-2008 to 24-02-2009; group 3, 02-02-2010 to 01-04-2010; and group 4, 18-02-2011 to 15-04-2011. The inter-observer reliability of the general scan-taking was checked during a 2-h simultaneous observation of group 4 by two observers. To this end, general proximity scans were made every 5 min. When the exact poles for all subjects in 24 scans of the two observers were compared, the agreement was marginal (kappa = 0.64, index of concordance = 0.66). However, if scoring two adjacent poles is counted as an agreement (which corrects for situations where an individual is sitting equidistantly between two poles, or moves from one pole to another during scoring), the agreement was amply sufficient (index of concordance = 0.82). Sexual behaviours were recorded ad libitum. For long-tailed macaques, which have only single-mount copulations, copulations were scored if they involved three or more thrusts. For rhesus macaques, which have multiple-mount copulations, only the first mount within a mount series was used for analysis. The remainder of the sexual behaviours (sexual present, taillift, waistgrasp and mount) were, for both species, in conformity with the ethogram of our previous studies (Overduin - de Vries et al. 2012, 2013). One or two observation sessions of typically 2 h each, and only occasionally 1 h (mean = 2.0, SD = 0.06), were conducted per day, resulting in respectively 160, 192, 142 and 73 h of ad libitum sampling for the four study groups.

### Screens providing hiding locations

We provided screens on a temporary basis and at different locations, so the animals could not use operant conditioning to copulate at a particular spot but had to be flexible when using the screens. The screens were provided to the monkeys just before the start of the first observation session of a given day. Each screen remained at a particular location for 24 h, except when mentioned otherwise. All groups had access to two screens at the same time, precluding the simultaneous monitoring of both sides of both screens by a single bystander monkey, while the screens were oriented along the line of sight of the human observer. The two screens were semi-randomly alternated between six possible locations.

Within the rhesus macaque groups, four different types of screens were created: (a) fully opaque, (b) transparent bottom, opaque top, (c) opaque bottom, transparent top, and (d) fully transparent. The dimensions of the screens (width × height) were 1.2 × 1.3 m. During each day, two different screens were simultaneously available to the monkeys. These screens were used during 42 observation days in group 1 and for 21 observation days in group 2. The screens in group 2 were replaced by two equal, fully opaque wooden screens measuring 2.8 × 1.13 m for further 31 observation days. These larger screens in group 2 were not removed after 24 h but remained in the group permanently.

Since the rhesus macaques hardly ever used the screens, we simplified the set-up of the experiment for the long-tailed macaque study and used only the screen type we presumed to be most effective. In group 3, two equal, fully opaque screens, measuring 3.0 × 0.8 m, were hung from the ceiling. Each of the screens was equipped with a sitting beam on each side of the screen. In addition, several equally elevated beams were provided without screens. This way, a possible preference for the screens would not be caused by a preference for elevated beams.

Since the fully opaque screens in group 3 were hardly used, we adjusted the set-up for the possibility that the macaques required a larger enclosure to show hiding behaviour and/or preferred another screen type that allowed monitoring of the audience while hiding. For group 4, one of the screens was similar to those used in group 3, but we added one peek-hole screen. This peek-hole screen measured 3.0 × 0.8 m and had 51 holes of 5-cm diameter. Both screen types were simultaneously available to the monkeys.

### Recording locations

To calculate inter-individual distances, we took scans under two different conditions: (1) ‘baseline behaviour’, in which the location of all animals during non-sexual behaviour is measured, and (2) ‘sexual behaviour’, in which the location of the animals involved in sexual behaviour, and the location of the alpha male are measured. The effect of the alpha male is expected to be strongest, since reported disruptions almost exclusively involve higher ranking individuals affecting lower ranking ones (Chapais 1983; Manson 1996). Moreover, in previous studies, we found that the alpha male has the strongest audience effect on its group members’ sexual behaviour (Overduin - de Vries et al. 2012, 2013). Therefore, the method used in the rhesus macaque study only determined the location of the alpha male in relation to the copulating couple. However, since we found audience effects of other individuals in our previous study, we decided to adjust the method in the subsequent long-tailed macaque observations, so that the location of all sexually mature group members could be determined under both conditions.

Baseline scans were taken at the start and end of each observation session for rhesus macaques, and every half hour for long-tailed macaques. All rhesus macaque scans consisted of individual locations indicated on a scaled map. All long-tailed macaque scans were taken by noting down for each individual the nearest pole of the enclosure. Each cage contained 0.22 vertical poles/m^2^. In addition, a monkey was noted as hiding near a screen if it was a rhesus macaque within 1 m from the screen or if it was a long-tailed macaque positioned on one of the beams attached to the elevated screen.

### Analysis

The strength of the effect of a particular bystander on the positioning of its group members’ sexual behaviour was calculated by taking the mean difference between baseline and sexual behaviour in distance from a particular bystander. This mean difference was taken as the dependent variable in a general linear model (GLM), and corrected for repeated measures from the same individuals, by incorporating male and female identity as random factors in the model. The estimated marginal mean resulting from the general linear model is hereinafter termed the ‘audience effect strength’.

For long-tailed macaques, we matched group cohesion between sexual and baseline behaviour (this was impossible for rhesus macaques, because we did not have data on group cohesion during sexual behaviour for this species). For each sexual behaviour scan, the mean inter-individual distance (MID) of the individuals that were not involved in sexual behaviour was calculated. For each sexual scan, a baseline scan was selected which had the nearest MID value. Subsequently, the distance of the copulating couple in the sexual scan and the selected baseline scan were compared. A single baseline scan was included in the analysis one to four times, but baseline scans were only repetitively included if both individuals involved in sexual behaviour in the matched sexual scans differed. Hence, each comparison of distance between sexual and baseline scan is unique.

Most tests were conducted using the software package ‘R’ version 2.10. The GLM analyses were executed using SPSS version 20. All statistical tests were two-tailed with α set at 0.05.

## Results

### The use of screens

We recorded 665, 80, 91 and 129 sexual interactions in groups 1, 2, 3 and 4, respectively, involving non-alpha males with females. In group 2, 25 of these interactions were observed when we had placed a permanent large screen. Individuals seldom copulated near any kind of screen (3 out of 745 for rhesus and 11 out of 220 for long-tailed macaques). Altogether, sexual interactions near screens did not occur significantly more often on the hidden side (6) than on the side visible to the alpha male (8) (lumping all data: binomial test: *P* = 0.79). Placing the extra large screen permanently in the group for 1 month (group 2) yielded one sexual interaction near the screen out of the 25 total sexual interactions in this period. The number of sexual interactions near screens was too low to conduct subsequent analysis on screen-type preferences.

### Distance

#### Rhesus macaques

Male rhesus macaques were positioned at significantly larger baseline distances from the alpha male than females (Kolmogorov-Smirnov test: *D* = 0.56, *N*_1_ = 9, *N*_2_ = 20, *P* < 0.05). During sexual behaviour, females were at significantly larger distances from the alpha male than baseline (Wilcoxon signed-ranks test: *V* = 2, *N* = 18, *P* < 0.0001, Fig. 1b). Likewise, males were at significantly larger distances from the alpha male during sexual behaviour than baseline (Wilcoxon signed-ranks test: *V* = 0, *N* = 9, *P* < 0.005, Fig. 1a).

#### Long-tailed macaques

Male long-tailed macaques had larger baseline distances from the alpha male than females (Kolmogorov-Smirnov: *D* = 0.73, *N*_1_ = 6, *N*_2_ = 15, *P* < 0.01). Without the correction for group cohesiveness (hence with a similar analysis as the rhesus macaques), both males (Wilcoxon signed-ranks test: *V* = 6, *N* = 10, *P* < 0.05, Fig. 1c) and females (Wilcoxon signed-ranks test: *V* = 22, *N* = 30, *P* < 0.0001, Fig. 1d) were farther away from the alpha male during sexual than during non-sexual behaviour. Similarly, after correcting for group cohesiveness, both females (GLMM: constant = 5.22, *N* = 30, *T* = 7.80, *P* < 0.0001) and males (GLMM: constant = 2.27, *N* = 10, *T* = 3.38, *P* < 0.001) were farther away from the alpha male during sexual than during non-sexual behaviour.

The audience effect strength of a (female or non-alpha male) bystander on the locations of females during sexual behaviour in group 3 was significantly dependent on the bystander’s rank, but not on the bystander’s baseline distance from the alpha male (Table 1). In group 4, the audience effect strength of a (female or non-alpha male) bystander on the locations of females during sexual behaviour was significantly dependent on the bystander’s general distance from to the alpha male, but not on the bystander’s dominance rank (Table 1).

**Table 1:** Factors influencing the audience effect strengths of non-alpha individuals in long-tailed macaque groups 3 and 4

Group	Parameter	Estimate	Std. error	*t*	*P* value
3	Intercept	6.71	1.75	3.83	0.0013*
	Proximity to alpha	−0.18	0.15	−1.23	0.23
	Dominance rank	−0.2	0.05	−3.69	0.0018*
4	Intercept	5.91	1.5	3.94	0.00096*
	Proximity to alpha	−0.64	0.21	−3.08	0.01*
	Dominance rank	0.01	0.03	0.4	0.69

The audience effect strength of non-alpha individuals was explained by the individual’s proximity to the alpha male and dominance rank. Statistics of a general linear model are provided with the audience effect strength of a particular individual on female distances as the dependent variable, and baseline proximity to the alpha male and dominance rank as predicting factors. *P* values smaller than alpha = 0.05 are marked (*)

## Discussion

Sneaky mating has been documented in rhesus and long-tailed macaques (Ruiz de Elvira and Herndon 1986; Overduin - de Vries et al. 2012, 2013), and in our study, we investigated which cognitive mechanisms may underlie these behaviours. We found that none of the macaques in the four observed groups systematically used opaque objects to hide sexual behaviour. Instead, both macaque species and both sexes distanced themselves from the alpha male, indicating that they created peripheral positions and employed TD level 1 for sneaky mating.

### Hiding of sexual behaviour

In contrast with earlier research (Gygax 1995), non-alpha males and females in none of the four observed groups systematically used opaque objects to hide sexual behaviour. Even when the screens were fully opaque, relatively large and permanent (group 2, 31 days), the monkeys rarely showed sexual activity near screens; moreover, this rare activity occurred with equal frequency on the visible and on the invisible side of the screen from the perspective of the alpha male. These results may be due to the high risk of discovery of monkeys hidden behind a screen when there is limited space. However, our results were similar in the group that had a double-sized enclosure (group 4 vs group 3) and therefore ample space. Alternatively, certain characteristics of our screens, however, may not have been optimal for the monkeys. The lack of sexual behaviour near screens did not reflect a fear of these relatively novel objects, since the monkeys used the screens to sit on and played around them. Moreover, a long period with permanent screens, allowing for habituation, did not result in the monkeys’ using them to hide sexual behaviour. Therefore, the most plausible explanation for our results is that macaques do not make use of (semi-)opaque objects to hide their sexual behaviour. This contradicts earlier results (Gygax 1995), but in that study, macaques may have used opaque objects for reasons other than restricting visibility, such as restricting accessibility, or because of their elevated or peripheral locations, which were avoided in our study’s set-up. Our results therefore eliminate strategies 3 to 6.

There is evidence however for the use of another strategy, *creating peripheral locations* (strategy 2), by both males and females. Non-alpha males of both rhesus and long-tailed macaque groups occupied more distant locations from the alpha male during non-sexual behaviour compared to females. This is consistent with a peripheral positioning of non-alpha males in wild macaque groups (Drickamer 1974; Berard et al. 1994; Hayakawa 2007; Inoue and Takenaka 2008; Dubuc et al. 2011). A peripheral location of non-alpha males may result in sneaky mating when females willing to copulate approach these males (no TD, strategy 1), as reported in rhesus and Japanese (*Macaca fuscata*) macaques (Berard et al. 1994; Hayakawa 2007). In this way, no active separation by the non-alpha male and female from bystanders is necessary for sneak copulations. However, in our study, not only females but also non-alpha male macaques had a larger distance from the alpha male during sexual behaviour than baseline. Therefore, sneaky mating in both rhesus and long-tailed macaques was consistent with strategy 2: creating distant locations. This was found in the general analyses (cf. le Roux et al. 2013) and when correcting for activity levels. Evidently, if females seek sexual behaviour with non-alpha males at the periphery of the group, both the female and non-alpha male move away even farther from the alpha male before they engage in the behaviour. Creating peripheral locations was found in both macaque species and both sexes, irrespective of their differences in despotism (Thierry 2007) and mounting behaviour (multiple vs single mounters: Shively et al. 1982) and the potential difference in motivation between males (Wilson 1981) and females (Ruiz de Elvira and Herndon 1986; Manson 1996). This is consistent with earlier research showing that both species and sexes more often initiate sexual behaviour at locations hidden from the alpha male (Overduin - de Vries et al. 2012, 2013) and expose themselves to the risk of aggression if discovered (rhesus macaques: Wilson 1981; Ruiz de Elvira and Herndon 1986; Manson 1996). Therefore, sneaky mating in both rhesus and long-tailed macaques results from active distancing from the alpha male (strategy 2: *creating peripheral locations*), consistent with TD level 1. In other species, the inhibition of copulation calls (geladas: le Roux et al. 2013), the uttering of alarm calls to maintain proximity (topi antelopes, *Damaliscuslunatus*: Bro-Jørgensen and Pangle 2010), and differential body coloration, with a sexual colour on the side of the female and a neutral colour on the side of the male (mourning cuttlefish, *Sepia plangon*: Brown et al. 2012), may represent tactical deception as a sexual strategy. While these strategies may be specific to these taxa or species, creating peripheral locations has general applicability and may be found in many species.

In long-tailed macaques, peripheral locations were also sought in response to non-alpha males. This depended on two different variables: on the bystander’s proximity to the alpha male (group 4), suggesting that the effects of non-alpha males were an epiphenomenon of the alpha male’s effect, and on the bystander’s dominance rank (group 3), indicating that, besides the alpha male, other high-ranking individuals affect the location of sexual behaviour of group members. The latter finding is consistent with audience effects from non-alpha individuals found in rhesus macaques (Overduin - de Vries et al. 2012) and long-tailed macaques (Overduin - de Vries et al. 2013), and with disruptions of copulations by non-alpha macaques that outrank one of the copulating partners (Niemeyer and Anderson 1983; Niemeyer and Chamove 1983; Manson 1996).

Sneaky mating does not necessarily involve hiding. By making use of a compartment outside the view of dominant males in rhesus and long-tailed macaques (Overduin - de Vries et al. 2012, 2013), sneaky mating may be achieved using the tactic of creating peripheral locations. Also, sneaky mating in free-ranging and wild populations, attributed to hiding behind opaque objects (Berard et al. 1994; Hayakawa 2007), may result from creating peripheral locations rather than specifically selecting concealing objects. In the wild, the vegetation or the landscape may provide multiple opaque objects. Increasing distance can make copulations less conspicuous since the probability is greater that opaque objects will block the view of the alpha male. Indeed, rhesus macaques at Cayo Santiago show sneaky mating in the periphery of the group (Berard et al. 1994). Therefore, copulating behind a subterfuge or down the cliff (Berard et al. 1994) may be achieved by simply increasing distance. Moreover, a relatively small increase in distance may be enough to guarantee sexual opportunities, as shown in wild geladas (le Roux et al. 2013). This is similar to the additional distance of about 5 m in our study (Fig. 1). Altogether, creating peripheral locations (TD level 1) may be effective in improving sexual opportunities by decreasing salience and also by increasing the chance objects will obscure the view of more dominant competitors.

### Tactical deception

Our results indicate that sneaky mating in macaques results from TD level 1. No evidence for TD level 1.5 or higher was found, because neither rhesus nor long-tailed macaques systematically hid sexual behaviour behind one of the provided screens. Therefore, they did not show an understanding of the visual perspectives of others (VPT). This contrasts with our study on long-tailed macaques that showed VPT in a paradigm involving food competition (Overduin - de Vries et al. 2014). There are three possible explanations for this discrepancy: (1) lack of motivation, (2) passive withholding or active manipulating of information and (3) complexity of the social situation.

First, the risk of receiving aggressive punishment in the sexual context may be lower than in the food context. Aggressive harassment of unconcealed sexual behaviour was seldom observed in this study (unpublished data). Other macaque studies report sexual harassment more consistently (Wilson 1981; Niemeyer and Anderson 1983), yet aggression during these harassing interruptions is rare (Wilson 1981). Despite the absence of aggressive harassment, less severe harassment or disruption by the mere presence of individuals, which is more difficult to observe, may occur more frequently within this and other studies, and may be the motive for concealing sexual behaviour. Besides, in our previous studies involving visually separated compartments, the alpha male reacted aggressively to copulating couples within his view (i.e. in the same compartment) in half of the cases for rhesus macaques (Overduin - de Vries et al. 2012), and a quarter of the cases for long-tailed macaques (Overduin - de Vries et al. 2013). The percentage of aggressive sexual harassment is equally high when compared to the food context: the percentage of aggression in response to unconcealed snatching of food [52 % of unconcealed attempts received aggression (Overduin - de Vries et al. 2014)]. Altogether, the motivation to use these cognitively demanding strategies may be similar in the food and in the sexual context and cannot explain the lack of evidence for TD level 1.5.

Second, using the provided screens for hiding sexual behaviour may require TD level 1 (strategies 3 and 4), level 1.5 (strategy 5) or level 2 (strategy 6). What level of TD is employed depends on how the actors withhold information from the bystander: passively withholding or actively manipulating information.

When passively withholding information, an individual refrains from particular acts in the presence of the bystander. This has been found in several monkey and ape species (e.g. mangabeys, *Cercocebus torquatus*: Coussikorbel 1994; chimpanzees: Menzel 1974). Similarly, passive withholding of information may explain how both rhesus and long-tailed macaques refrain from sexual behaviour in the presence of dominant individuals (Overduin - de Vries et al. 2012, 2013). Refraining from certain behaviour may only require knowledge that performing the act may lead to aggression or to a resource loss, and not reflect an understanding of VPT. Similarly, hiding behind screens does not necessarily require VPT. Monkeys may learn by operant conditioning to passively refrain from sexual behaviour when away from the screens (strategies 3 and 4). However, the number of sessions we administered might not have been enough to allow monkeys to learn by operant conditioning that the screens prevent punishment.

Actively manipulating information at TD level 1.5 may occur when an animal coincidentally ends up behind a screen, notices that it is hidden, and subsequently learns to actively go to the screen for sexual activity. The initial chance of coincidental hiding may have been too low in our study. To increase the frequency of ending up behind a screen, an animal may purposefully go to the screen with the goal of hiding sexual behaviour, i.e. TD level 2. However, this requires more complicated cognitive skills. The animal has to be able to imagine itself in a situation that does not exist at that time, that is, be capable of so-called ‘self-projection’ (Buckner and Carroll 2007). Therefore, active manipulating requires a TD 2 level and is almost impossible for animals only using TD at level 1.5. Indeed, the capacity has never been tested in monkeys and even apes are not capable of understanding imagined situations (Call and Tomasello 1999). Therefore, the absence of the use of screens may be explained by an absence of the required cognitive skills.

Third, VPT may only be used in socially less complex situations, such as food competition, that only involve one concealing individual, but not in more complex situations, like sneaky mating, that require the concealment of behaviour by two individuals simultaneously. This explanation counters the social intelligence hypothesis (Humphrey 1976), which predicts that socially complex situations drive the evolution of cognitive capacities. This hypothesis predicts that when VPT is evolved to cope with cognitively complex situations, such as sneaky mating, it should be used in socially complex situations and may also be used in a relatively simple social context (food competition). However, our study found the opposite. If this interpretation is correct, our results challenge the social intelligence hypothesis.

In summary, our results indicate that sneaky mating by rhesus and long-tailed macaques is not accomplished by seeking locations near or behind opaque objects (strategies 3–6). Instead, both non-alpha males and females actively increase their distance from the alpha male before engaging in sexual behaviour (strategy 2). The inhibiting effect of bystanders was strongest for the alpha male, and dominance-dependent sexual competition appears to be an important factor in the sexual dynamics of macaques. The mechanism used to conceal sexual behaviour involved TD level 1 and not TD level 1.5. This indicates that a reduction of sexual competition is not necessarily accomplished by high cognitive mechanisms like VPT. Our results show that sneaky mating in macaques probably results from operant conditioning to increase the distance from bystanders during sexual interactions. Therefore, TD level 1 leading to sneaky mating can be adopted by any species that is able to flexibly apply operant conditioning and wherever hiding copulations results in benefits for the agent.

## Electronic supplementary material

ESM 1 Table 1. The different types of screens, number of different types of screens and number of possible and simultaneously used locations for screens provided to the rhesus (group 1 and 2) and long-tailed (group 3 and 4) macaques. A particular constellation of screens was provided for 24 h, only in group 2 during the second part of the study were two screens permanently (31 days) provided. (DOCX 63 kb)
