# Supplementary material for: Tactical deception to hide sexual behaviour: macaques use distance, not visibility
Source: Behav Ecol Sociobiol. 2015 Jun 5;69(8):1333–42. doi: 10.1007/s00265-015-1946-5 (PMC4521099; doi:10.1007/s00265-015-1946-5)
Supplement: Supplementary file 1 — Table 1. The different types of screens, number of different types of screens and number of possible and simultaneously used locations for screens provided to the rhesus (group 1 and 2) and long-tailed (group 3 and 4) macaques. A particular constellation of screens was provided for 24 h, only in group 2 during the second part of the study were two screens permanently (31 days) provided. (DOCX 63 kb) [file 265_2015_1946_MOESM1_ESM.docx]

Tactical deception to hide sexual behaviour: macaques use distance, not visibility

Behavioral Ecology and Sociobiology

A.M. Overduin – de Vries

B.M. Spruijt

H. de Vries

E.H.M. Sterck

Corresponding author:

E.H.M. Sterck

Animal Ecology, Utrecht University

E.H.M.Sterck@uu.nl

| **group** | **screen types** | | | |  | **number of different screens** | **number of possible screen locations** | **number of screens simultaneously available** | **how long screens remained on the same location** |
| --- | --- | --- | --- | --- | --- | --- | --- | --- | --- |
|  | full opaque | bottom see trough top opaque | bottom opaque top see through | full see trough | peak hole |  |  |  |  |
| 1 | x | x | x | x |  | 4 | 6 | 2 | 24 h. |
| 2 | x | x | x | x |  | 4 | 6 | 2 | 24 h. |
| 2 | x |  |  |  |  | 1 | 2 | 2 | 31 days |
| 3 | x |  |  |  |  | 1 | 6 | 2 | 24 h. |
| 4 | x |  |  |  | x | 2 | 6 | 2 | 24 h. |
